# Timely Diagnosis of Acute Lymphoblastic Leukemia Using Artificial Intelligence-Oriented Deep Learning Methods

**DOI:** 10.1155/2021/5478157

**Published:** 2021-11-11

**Authors:** Sorayya Rezayi, Niloofar Mohammadzadeh, Hamid Bouraghi, Soheila Saeedi, Ali Mohammadpour

**Affiliations:** ^1^Department of Health Information Management and Medical Informatics, School of Allied Medical Sciences, Tehran University of Medical Sciences, Tehran, Iran; ^2^Department of Health Information Technology, School of Allied Medical Sciences, Hamadan University of Medical Sciences, Hamadan, Iran; ^3^Clinical Research Development Unit of Farshchian Heart Center, Hamadan University of Medical Sciences, Hamadan, Iran; ^4^Health Information Management Department, School of Allied Medical Sciences, Tehran University of Medical Sciences, Tehran, Iran

## Abstract

**Background:**

Leukemia is fatal cancer in both children and adults and is divided into acute and chronic. Acute lymphoblastic leukemia (ALL) is a subtype of this cancer. Early diagnosis of this disease can have a significant impact on the treatment of this disease. Computational intelligence-oriented techniques can be used to help physicians identify and classify ALL rapidly. *Materials and Method*. In this study, the utilized dataset was collected from a CodaLab competition to classify leukemic cells from normal cells in microscopic images. Two famous deep learning networks, including residual neural network (ResNet-50) and VGG-16 were employed. These two networks are already trained by our assigned parameters, meaning we did not use the stored weights; we adjusted the weights and learning parameters too. Also, a convolutional network with ten convolutional layers and 2*∗*2 max-pooling layers—with strides 2—was proposed, and six common machine learning techniques were developed to classify acute lymphoblastic leukemia into two classes.

**Results:**

The validation accuracies (the mean accuracy of training and test networks for 100 training cycles) of the ResNet-50, VGG-16, and the proposed convolutional network were found to be 81.63%, 84.62%, and 82.10%, respectively. Among applied machine learning methods, the lowest obtained accuracy was related to multilayer perceptron (27.33%) and highest for random forest (81.72%).

**Conclusion:**

This study showed that the proposed convolutional neural network has optimal accuracy in the diagnosis of ALL. By comparing various convolutional neural networks and machine learning methods in diagnosing this disease, the convolutional neural network achieved good performance and optimal execution time without latency. This proposed network is less complex than the two pretrained networks and can be employed by pathologists and physicians in clinical systems for leukemia diagnosis.

## 1. Introduction

Leukemia is a type of cancer that affects the bone marrow and is divided into four main categories: acute lymphoblastic leukemia (ALL), acute myeloid leukemia (AML), chronic lymphoid leukemia (CLL), and chronic myeloid leukemia (CML) [1, 2]. Acute lymphoblastic leukemia is a type of cancer that affects the lymphocytes and lymphocyte-producing cells in the bone marrow and is the second most common acute leukemia in adults [3]. ALL is also the most common cancer in children, and although it is treated in 90% of children, it remains still one of the leading causes of death in children and adults [4].

If ALL is diagnosed earlier, the treatment process will be immediate, and the patient survival rate will increase significantly. Depending on the patient's symptoms and level of risk, various treatment options are available, including chemotherapy, radiotherapy, anticancer drugs, or a combination of these [5]. Various treatment options can reduce the disease's symptoms or lead to a complete cure for the patient. These treatment options significantly increase survival and improve patients' quality of life [6].

Correct diagnosis is crucial in choosing the type of treatment (radiation therapy, medication, and chemotherapy) and planning. All the methods used to diagnose leukemia are manual, which depends entirely on trained and experienced medical professionals. Microscopic evaluations of blood cells are an essential step in the diagnosis. This assessment requires an expert pathologist to identify abnormal patterns in blood cells. Medical images are one of the most important diagnostic tools in the field of medicine. Because the information in these images is hidden in the pixels, the images cannot be used without analysis and interpretation; therefore, they need to be processed [7]. Unfortunately, the possibility of errors in the interpretation of medical images is not negligible; tissue and cell diversity, lack of specialists, long working hours, eye fatigue, and lack of concentration are some of the factors that negatively affect the accuracy of the diagnosis [8]. Extensive research has been done today to mechanize medical images' interpretation to limit these problems. Computer-assisted image analysis can be practical in the early detection of diseases [9, 10].

Due to recent advances in pathological and microscopic image analysis, algorithms and techniques have been developed for clinical diagnosis and evaluation. Recently, artificial intelligence has shown remarkable success in analyzing medical images due to the rapid development of deep learning and machine learning algorithms. These successes have increased the power to solve complex real-world problems and image analysis [11]. Machine learning is one of the most important topics in the field of medicine due to its role in improving diagnosis and sensitivity in classifying diseases; however, some researchers consider feature engineering to be a significant weakness of machine learning [12]. In other words, feature extraction methods are often designed manually and then applied to the image. Deep learning eliminates the weakness of feature engineering and is a subset of machine learning methods that, by automatically extracting features from the image and increasing the system's accuracy, make it possible to solve very complex problems to a large extent. However, these networks learn features directly from the input data [7].

Due to the multiple challenges in diagnosing acute lymphoblastic leukemia, pathological examination of blood slides by intelligent algorithms can help physicians definitively diagnose, and pathologists can use these methods to analyze microscopic images and treatment measures. Therefore, this study aims to provide accurate and immediate diagnosis and classification of acute lymphoblastic leukemia by processing patients' microscopic images. This diagnostic approach will be based on deep learning and machine learning techniques. In other words, by developing and designing image classification models, the distinction between normal and cancer cells will be identified. Although the purpose of these research studies is achieving high accuracy, using types of parameters and hyperparameters to assess their role in network training is important issue. Therefore, we proposed these networks to evaluate and compare different networks based on their architecture and their parameter.

### 1.1. Our Contribution

The main contributions of this study are listed as follows:

- A large number of pathological images related to acute lymphoblastic leukemia and healthy cells are chosen.
- The preprocessing methods such as resizing, segmenting, and normalizing algorithms are applied to prepare the chosen dataset.
- Extracted critical features are used to classify acute lymphoblastic leukemia and healthy cells by two pretrained networks, i.e., ResNet-50 and VGG-16 and a novel proposed convolutional network. Six common machine learning techniques are also utilized.
- Our proposed convolutional network has a simpler architecture than employed pretrained networks.
- We have also trained pretrained networks with our assigned parameters, i.e., we have not used the stored weights in these networks, and only their architectures were considered in our study.
- Validation accuracies of developed network and pretrained ones are optimal. Accuracy of ResNet-50, VGG-16, and proposed convolutional network is approximately 81.63%, 84.62%, and 82.10%, respectively, and areas under the receiver operating characteristic (AUROC) curves are 0.9 or 0.8. Performance analysis suggests a renovation of our proposed approaches by comparing related works.

### 1.2. Related Works

A study conducted by Thanh et al. in 2018 aimed to establish a decision support system for the early diagnosis of patients with acute leukemia in the early stages of the disease for treatment. The focus of this study was on the diagnosis of acute myeloid leukemia. This paper proposed a convolutional neural network-based method containing seven layers for detecting normal and abnormal images of blood cells. This method, with a dataset containing 1188 images of blood cells, had an accuracy of 96.6. The first five layers perform feature extraction, and the other two layers (fully connected and softmax) classify the extracted features [13].

In another research conducted in 2018 by Shafique and Tehsin, a deep convolutional neural network (CNN) has been used for automatic diagnosis of ALL and classification of its subgroups into four classes, namely, L1, L2, L3, and normal. AlexNet has been adopted for training; the last layers of the trained network have been replaced with new layers that can classify the input images into four classes. To reduce overtraining, the data augmentation method has been developed. For the diagnosis of ALL, the sensitivity was 100%, the specificity was 98.11%, and the accuracy was 99.50%. Moreover, for the classification of ALL subtype, the sensitivity was 96.74%, the specificity was 99.03%, and the accuracy was 96.06% [14].

A new method for detecting different types of leukemia from microscopic images of blood cells using convolutional neural networks has been considered in a study by Ahmed et al. The effects of data augmentation on an increasing number of combined training samples have also been investigated. The study used two publicly available leukemia data sources: ALL-IDB and ASH Image Bank. In the next step, seven different image transformation methods are used as data augmentation. A CNN architecture is then designed that can detect all subtypes of leukemia. Also, popular machine learning algorithms such as Naive Bayes, support vector machine, k-nearest neighbor, and decision tree have been used; 5-fold cross-validation has been applied to evaluate performance. The results showed that the CNN model's performance was 88.25 and 81.74% in the patient and healthy groups, respectively. This study shows that the CNN model performs better than popular machine learning algorithms [15].

## 2. Materials and Methods

### 2.1. Data Acquisition

**Figure 1:**
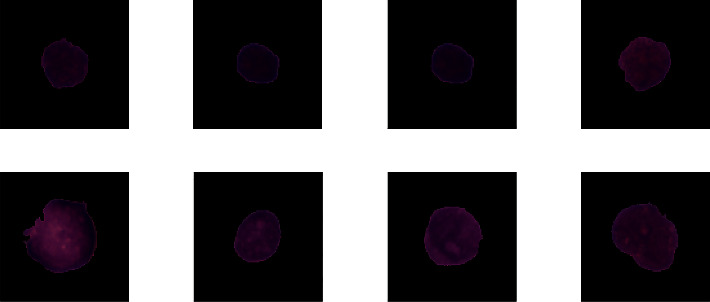
Sample of cell images: (a) healthy and (b) leukemic.

The dataset used in this study was collected from a CodaLab competition to classify leukemic cells from normal cells in microscopic images. The dataset contains images of leukemic B-lymphoblast cells (malignant cells) and normal B-lymphoid cells [16]. The dataset has been preprocessed as cells have been normalized and segmented from the original images. The images have a final size of roughly 300 × 300 pixels. Nevertheless, for this study, we changed the images size to 70 × 70. The complete dataset is composed of images from 118 patients. In each folder, there are cell images from each patient. After images preprocessing, the dataset contains a total of 73 patients, of which 47 have cancer and 26 are healthy. The separation of images in training and testing will be done by images of malignant and healthy cells instead of by patients. By doing so, we mix images from the same patient in the training and testing. Finally, 12,528 images which included 4,037 healthy and 8,491 leukemia cells were used to train networks. Given that the applied dataset is imbalanced, the number of images related to cancer cells is more than the number of images related to healthy cells. In coding, we used the weighting method, which is a standard way to overcome data imbalances. In this technique, the effect of data in the training process is based on its number. This indicates that the weight of a class with more data is less than the weight of a class with fewer data. So finally, all data have the same effect, and obtained results are reliable. However, the difference in weights will affect the classification of the classes during the training stage; the chief purpose is to penalize the misclassification made by the minority class by setting a higher-class weight and at the same time reducing weight for the majority class.  Figure 1 shows images of a healthy cell and leukemic cell from the dataset.

### 2.2. ResNet-50 Neural Network

**Figure 2:**
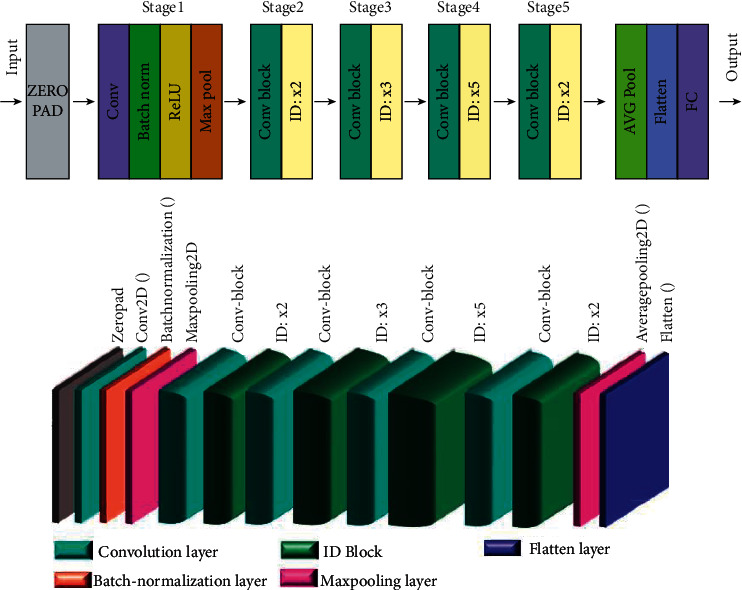
The architecture of the ResNet-50 network.

Residual neural network (ResNet) is a type of convolutional neural network proposed by Microsoft Corporation that won first place in the ILSVRC 2015 competition. With a depth of 152 layers, this architecture was named the deepest at that time. The network consists of multiple residual modules stacked upon each other to form the main building block of ResNet architecture. The residual module has two options; it can either perform a series of operations on the input or skip all of them. These stacked residual modules fit a complete network [17].  Figure 2 describes this neural network architecture. “ID BLOCK” in the diagram stands for “Identity block,” and “ID BLOCK × 3” means stack 3 identity blocks together.

The details of this ResNet-50 model are as follows:

(i) Zero-padding pads the input with a pad of (3, 3).
(ii) Stage 1:
  - The 2D convolution has 64 filters of shape (7, 7) and uses a stride of (2, 2). Its name is “conv1.”
  - BatchNorm is applied to the channel's axis of the input.
  - Max-pooling uses a (3, 3) window and a (2, 2) stride.
(i) Stage 2:
  - The convolutional block uses three set of filters of size [64, 64, 256], “f” is 3, “s” is 1, and the block is “a.”
  - The 2 identity blocks use three set of filters of size [64, 64, 256], “f” is 3, and the blocks are “b” and “c.”
(i) Stage 3:
  - The convolutional block uses three set of filters of size [128, 128, 512], “f” is 3, “s” is 2, and the block is “a.”
  - The 3 identity blocks use three set of filters of size [128, 128, 512], “f” is 3, and the blocks are “b,” “c,” and “d.”
(i) Stage 4:
  - The convolutional block uses three set of filters of size [256, 256, 1024], “f” is 3, “s” is 2, and the block is “a.”
  - The 5 identity blocks use three set of filters of size [256, 256, 1024], “f” is 3, and the blocks are “b,” “c,” “d,” “e,” and “f.”
(i) Stage 5:
  - The convolutional block uses three set of filters of size [512, 512, 2048], “f” is 3, “s” is 2, and the block is “a.”
  - The 2 identity blocks use three set of filters of size [512, 512, 2048], “f” is 3, and the blocks are “b” and “c.”
(i) The 2D average pooling uses a window of shape (2, 2), and its name is “avg_pool.”
(ii) The flatten layer does not have any hyperparameters or name.
(iii) The fully connected (dense) layer reduces its input to two classes using a softmax activation.

**Figure 3:**
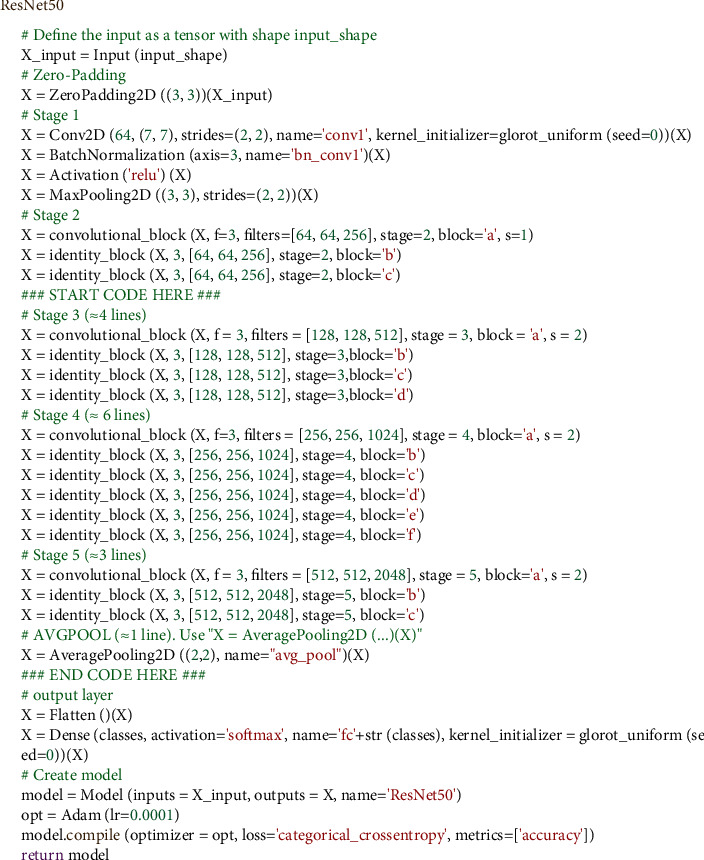
The stages of ResNet-50 in the form of pseudocode.

From the total number of 12,528 images, 80% (10022 numbers) were used as training data and 20% (2506 numbers) as test data. The activation for all layers except the last layer was ReLU function. Adam, with a learning rate of 0.0001, was selected for the optimization function. This network was trained over 100 training epochs, and data were transmitted to the network in batches of 16 size (batch-size). The duration of each epoch was 28 s. The stages of ResNet-50 in the form of pseudocode are given in Figure 3.

### 2.3. VGG-16 Neural Network

**Figure 4:**
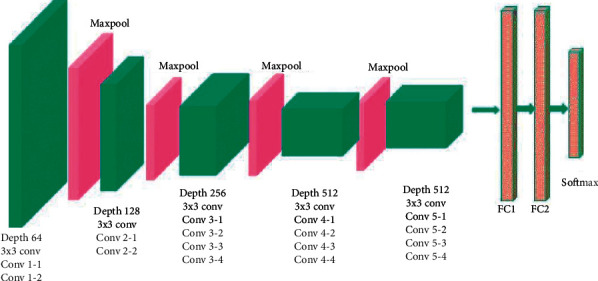
The architecture of the VGG-16 network.

It is also called the OxfordNet model, named after the Visual Geometry Group from Oxford. Number 16 refers that it has a total of 16 layers that have some weights. The architecture for VGG-16 network is shown in Figure 4. From the total number of 12528 images, 80% (10022 numbers) were used as training data and 20% (2506 numbers) as test data. The input to the main VGG-16 model is 224 × 224 × 3 pixels images. However, we changed it to 70 × 70 × 3. Then, there are two convolution layers with each 70 × 70 × 64 size, two convolution layers of 128 filter length, three convolution layers of 256 filter length, and three convolution layers of 512 filter length. Then again, there are three convolution layers of 512 filter length. The Kernel size is 3 × 3, and the pool size is 2 × 2 with step 2 × 2 for all the layers. The output of the last pooling layer is 2D which must be converted into a 1D layer so as to be sent to fully connected layers, done by a flatten layer. After the convolution layers, two 4096 fully connected layers and two fully connected layers were used to classify data into two classes by softmax activation function. The activation for all layers except the last layer was ReLU function. Adam, with a learning rate of 0.0001, was selected for the optimization function. Also, the same padding is used for all convolution layers. This network was trained over 100 training epochs, and data were transmitted to the network in batches of 32 size (batch-size). The duration of each epoch was 22 s.

### 2.4. Convolutional Neural Network

**Figure 5:**
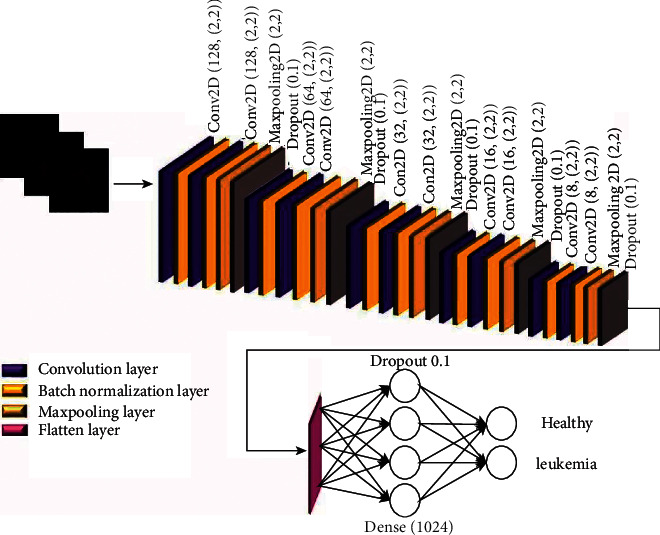
Architecture of the proposed convolutional neural network.

Convolutional neural networks (CNNs) are a critical class of deep learning methods in which multiple layers are strongly trained. These networks have very efficient and widespread applications in neural computer vision. In general, a CNN consists of three main layers: a convolution layer, a pooling layer, and a fully connected layer. Different layers function in different ways, which leads to ultimate learning. This network can be applied alone or alongside other networks for data classification [18]. The proposed architecture for the convolutional neural network is shown in Figure 5. Also, from the total data, 80% were used as training data and 20% as test data. The network includes two convolution layers of 128 filter length, two convolution layers of 64 filter length, two convolution layers of 32 filter length, two convolution layers of 16 filter length, and two convolution layers of 8 filter length. The 2*∗*2 kernel function was considered for all layers. In this architecture, the 2*∗*2 max-pooling layer is considered with strides 2. The output of the last pooling layer is 2D and is converted to a 1D layer by a flattened layer. The same padding has been used for all convolution layers. A 1024 fully connected layer and two fully connected layers have been used to classify data into two classes by softmax activation function after the flatten layer. In order to prevent overfitting, batch-normalization layers were considered after all convolution layers and dropout layers with a rate of 0.1 considered after all max-pooling layers. Adam, with a learning rate of 0.0001, was selected for the optimization function. This network was trained over 100 training epochs, and data were transmitted to the network in batches of 32 size (batch-size). The duration of each epoch was 14 s.

Our entire implementation is done in Keras with tensorflow backend. The networks in this research run on Google Colaboratory (Colab) environment. Colab provides a platform for running Python codes, especially machine learning, deep learning, and data analysis.

## 3. Experimental Results

As mentioned, the main purpose of this study was to classify cell images into healthy and leukemic classes. The dataset included 12,528 images of 4,037 healthy cells and 8,491 leukemic cells. One of the main criteria for evaluating artificial neural networks is network classification accuracy. By dividing data into training and test data, one accuracy for the training data and one for the test data are achieved during each cycle. The mean accuracy of training and test networks for 100 training cycles to classify into two classes of healthy and leukemia is calculated for the final evaluation of the network as validation accuracy. Also, common evaluation criteria such as precision, recall, and *F*-measure were calculated for three networks.  Table 1 summarizes the results of these three networks. The training accuracy of the ResNet-50 is found to be 95.76%, and validation accuracy is 81.63%. The training accuracy of the VGG-16 is 97.41%, and validation accuracy is 84.62%. Also, the training accuracy of the proposed convolutional network is 85.79%, and validation accuracy is 82.10%.

The precision, recall, and *F*-measure of two classes obtained from the ResNet-50, VGG-16, and convolutional neural network are summarized in Tables 2–4, respectively.

**Figure 6:**
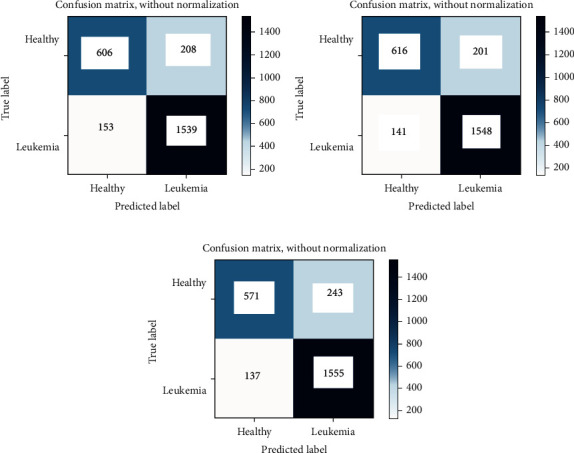
Confusion matrix analyses of the proposed model representing TP, TN, FP, and FN ratio obtained from the testing dataset of (a) ResNet-50, (b) VGG-16, and (c) proposed convolutional neural network.

A confusion matrix is needed to measure classification performance. In Figure 6, there are confusion matrices for ResNet-50, VGG-16, and proposed convolutional neural network models in test data. The test data include 814 healthy and 1,692 leukemia images. In the ResNet-50 model, the number of TP is 606 and FN is 208. In this pretrain CNN model, 153 out of 1,692 leukemia data were misclassified. When the results obtained by the VGG-16 model are examined, 616 images were classified correctly in the healthy class and 201 images were classified incorrectly. In this model, 141 out of 1,692 leukemia data were misclassified. Another network in the study is proposed CNN. For the metric evaluation of this network, 571 TP, 243 FN, 137 FP, and 1555 TN values were reached.

**Figure 7:**
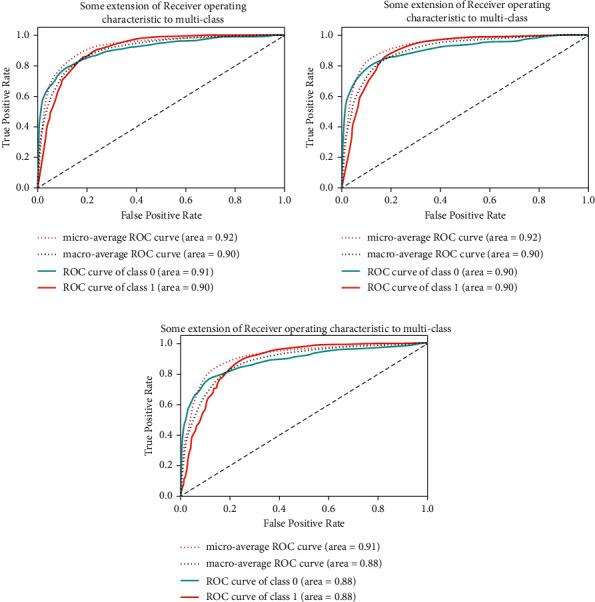
ROC plot of (a) ResNet-50, (b) VGG-16, and (c) convolutional neural network.

Figure 7 includes the receiver operating characteristic (ROC) value of pretrained CNN models and CNN model. The ROC curve area values of the ResNet-50 model for the healthy class are 0.91, and for leukemia, the class is 0.90. The 0.90 ROC curve area value is obtained using VGG-16 for both the healthy class and leukemia class. For the proposed CNN model, ROC curve area values were obtained 0.88 for both classes.

**Figure 8:**
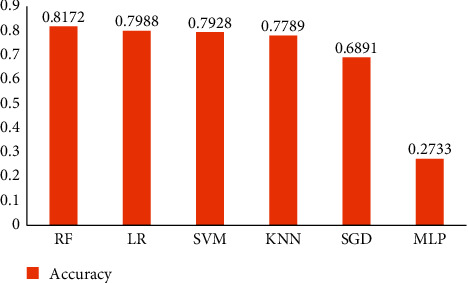
Comparison of the classification accuracy resulting from machine learning classifiers.

The use of machine learning (ML) algorithms that can learn from data without the need for explicit programming offers a promising avenue for meeting the high costs and shortages surrounding cell imaging. In this study, the accuracy obtained from machine learning classifiers such as support vector machine (SVM), nearest neighbor (NN), random forest (RF), stochastic gradient descent (SGD), logical regression (LR), and multilayer perceptron (MLP) was compared for classification into two classes (Figure 8). The obtained accuracy was 81.72% for RF, 79.88% for LR, 79.28% for SVM, 77.89% for KNN, 68.91% for SGD, and 27.33% for MLP.

The precision, recall, and *F*-measure for each set of healthy and leukemic cell images were calculated by these classifiers and are summarized in Table 5. For the healthy group, the highest precision of 83% was obtained with RF, the highest recall of 66% with KNN, and the highest *F*1-score of 68% with RF and LR. For leukemic, the highest precision of 83% was obtained with LR and KNN, the highest recall of 94% with RF, and the highest *F*1-score of 87% with RF. Comparison of proposed networks with other developed machine learning-based classifiers is presented in Table 6.

## 4. Discussion

In this study, the main objective was to develop three deep learning networks and famous machine learning techniques to classify acute lymphoblastic leukemia into two classes; the applied dataset contains leukemic B-lymphoblast images cells (malignant cells) and normal B-lymphoid cells. Finally, 12,528 images which included 4,037 healthy and 8,491 leukemia cells were utilized to train networks. The weighting technique has been utilized to overcome the problem of inequality in the number of data. In the present study, we developed a convolutional network for image classification. This convolutional network has a simpler architecture than pretrained networks, and due to the used parameters, its validation accuracy result is relatively less than the accuracy of VGG-16. Notably, in this study, we trained pretrained networks with new assigned parameters; in other words, we did not apply the stored weights in these networks and we adjusted the training parameters ourselves; in fact, we only used their architecture in our study to classify the images.

In [14], acute lymphoblastic leukemia detection was conducted using a pretrained AlexNet deep learning neural network. This network contains five convolutional layers with three max-pooling layers. Each convolutional layer in AlexNet architecture is followed by rectified linear unit (ReLU). Data augmentation technique to reduce overfitting was used. Acute lymphoblastic leukemia subtype classification's sensitivity was 96.74%, specificity was 99.03%, and accuracy was 96.06%. The highest accuracy obtained from this study (96.06%) with a value of 0.98 is less than the applied convolutional network (VGG-16) of our research for 100 training cycles. Our research's execution time was longer due to the complexity and more layers of the used networks (VGG-16 = 22 s for each epoch, ResNet = 28 s, and proposed CNN = 14 s), which justifies the good accuracy obtained [11]. For three networks, we applied several layers of convolution and created complex networks, which can be justified by the large volume of data at hand and the goal of increasing classification accuracy. Compared with our work that is done in Keras with tensorflow backend and runs on Google Colaboratory (Colab) environment, but in this study, the MATLAB environment has been used.

In another work [15], two open access data sources: ALL-IDB and the American Society of Hematology (ASH) Image Bank, were applied. Several image transformation approaches like rotation, flipping, and shifting were utilized to obtain different versions of original images. The CNN architecture contained different convolutional layers (32 feature map with the size of 3*∗*3), a max-pooling layer with the size of 2∗2, flatten layer, and fully connected layers with ReLU and softmax activation functions; they setup two types of optimizers such as SGD (stochastic gradient descent) and Adam optimizers one type at a time. The results obtained from experiments demonstrated that the proposed CNN model performance has 88.25% accuracy in leukemia versus healthy. This model in CNN has the third-best score, which is 88.25% in accuracy. Studies by Shafique and Tehsin [14] and Thanh et al. [13] outperformed this developed CNN model in the binary classification problem; considering they did not practice any cross-validation in evaluation, it is unknown how test and training sets were chosen.

In a work conducted by Kasani et al. [6], novel ensemble methods based on deep convolutional networks were applied. The researchers employed different data augmentation techniques like horizontal and vertical flips, contrast adjustments, and brightness correction to enlarge the dataset. Using data augmentation methods enables researchers to yield better performance compared with nonaugmented data. The obtained experimental results demonstrated that the proposed approach could fuse features extracted from the best deep learning models and outperformed individual networks with a test accuracy of 96.58% in leukemic B-lymphoblast detection. The chosen ensemble yielded a higher overall accuracy, of 96.58%; in contrast to our work, the computation time of the proposed architecture for each epoch during the training phase was approximately 130 min, while the execution time in our work is less than this. In our study, VGG-16 network performed the best compared with the proposed CNN and ResNet-50. The reason for the high accuracy of VGG-16 is its straightforward architecture. It has got two contiguous blocks of two convolution layers followed by a max-pooling, then it has three contiguous blocks of three convolution layers followed by max-pooling, and at last we have three dense layers. VGG-16 has a remarkable feature extraction capability so that it can obtain a good effect in image classification. VGG-16 has a better feature learning ability than ResNet-50 because it is deeper than ResNet-50, and it can get more sparse features [12]. A proposed deep learning-based hybrid approach, which is enriched by complex data augmentation procedures, is able to elicit high-level features from input images [19]. Experimental results confirm that the developed model yields better prediction than individual models [8, 20] for leukemic B-lymphoblast classification with 96.17% overall accuracy.

Another leukemia diagnosis technique was developed by Rehman et al. [8]. The proposed method using the AlexNet model with CNN is configured according to the selected data. Three last layers of this network are fully connected, and the classification layer is fine-tuned to the new layers of the classifier and is set according to the new data. Other classifiers like KNN, Naive Bayesian, and SVM are also checked for the performance evaluation. Experimental results show that the proposed network achieved 97.78% accuracy while support vector machine classifier reached 90.91% accuracy. In this work, experimental results from machine learning-based classifiers function revealed that random forest has achieved the maximum accuracy of 81.72% compared with other applied classifiers. Random forest is great with high dimensional data since we are working on subsets of data. It is faster than other techniques because we are working on a subset of main features in this method; this algorithm is robust to outliers and nonlinear data by essentially binning outliers [21]. Remarkably that because our used dataset was not very large, RF achieved optimal accuracy. Our methods were implemented on Python 2.7, based on the Keras library, and Adam optimizer was used too, while in the before mentioned work, the proposed architecture is implemented in MATLAB 2017a. Result analysis and comparison of previous works are shown in Table 7.

The developed networks in this study have the following advantages and disadvantages:

- The proposed convolutional network has a simpler architecture or less complexity than two pretrained networks.
- The execution time (the duration of each epoch) or the runtime of the proposed convolutional network is less than pretrained nets. Therefore, the use of ordinary hardware and memory can be enough to run our proposed convolutional network.
- By using the proposed CNN, a simpler architectural design with low latency can rapidly classify two classes of leukemia and healthy cells at the start of the development stage.
- If a relatively higher performance accuracy is considered, VGG-16 network which we have trained with our adjusted parameters can be applied. Nevertheless, the remarkable thing about ResNet-50 network is that it runs twice as long as the developed convolutional network. In systems with normal (not very strong) hardware and software specifications, the implementation of this network may be slightly delayed.
- However, there is a trade-off between high-performance accuracy and longer execution times that one can be chosen.
- The average accuracy (validation accuracy) in VGG-16 is more than that of the other two networks (ResNet and proposed CNN). Our proposed convolutional network has a relatively good average accuracy, which is higher than the performance accuracy of ResNet-50.
- It is true that the proposed network has less performance accuracy (validation accuracy) than VGG-16, but the simpler architecture, more efficient execution time, and the need for fewer computations will result in its use being considered.

## 5. Conclusion

One of the areas of adopting artificial intelligence and machine learning is in the health industry. Deep networks are currently being designed and developed to detect various diseases based on the images. In this paper, we investigated applications of deep CNNs in which we developed pretrained VGG-16 and ResNet for the diagnosis and classification of ALL and discerned between healthy and cancer cases. Furthermore, a novel CNN for this before mentioned target was configured. By performing image preprocessing techniques, we were able to reach 95.76% training accuracy of ResNet, 97.41% of VGG-16, and 85.79% of proposed CNN. In addition to the three applied deep networks adopted in this investigation, six machine learning techniques have been developed to classify ALL. In this study, the convolutional neural nets and machine learning techniques are used to understand deep features of images, and they can classify the images taking advantage to gain an increase in classification accuracy and precision. Our ultimate goal in this study was to compare the performance of three networks that we can use to classify based on priorities such as runtime, accuracy, architecture, or complexity. Pretrained networks are robust and have unique architecture, so it is quite expectable to obtain better results. Also, running time is an important criterion to evaluate neural networks. Pretrained networks often take a long time to run, so they are slow, but our proposed network was fast, and despite its simple architecture, obtained results were promising. The proposed approaches are assistance for the laboratory experts and pathologists, which has a tremendous clinical impact on leukemia patients. In our study, we have used a limited amount of training and evaluation dataset, which can affect the training process of deep neural networks. Therefore, we plan to configure deep learning to learn from scratch with larger image datasets in the future direction. These computational systems can be utilized in everyday life and help the specialist and oncologist detect leukemia effectively. Furthermore, the more leukemia images CNN can manage, the higher accuracy reached.

In future directions, consequently, the requirement for a large enough dataset to build an effective CNN architecture in leukemia diagnosis is exceptionally impressive. Because the used dataset was obtained from just one type of leukemia disease—the acute lymphoblastic leukemia (ALL)—we would prefer to practice a new dataset including four types of leukemia to assess our architectures.

## Figures and Tables

**Table 1:** Results from ResNet-50, VGG-16, and the proposed convolutional neural network for classification into two classes of healthy and leukemia.

Network	Training accuracy	Test accuracy	Train loss	Test loss
ResNet-50	0.9576	0.8163	0.1064	0.7592
VGG-16	**0.9741**	**0.8462**	0.0653	0.9236
Proposed convolution	0.8579	0.8210	0.3356	0.4517

**Table 2:** Precision, recall, and *F*-measure of ResNet-50.

	Precision	Recall	*F*-measure
Healthy	0.80	0.74	0.77
Leukemia	**0.88**	**0.91**	**0.90**

**Table 3:** Precision, recall, and *F*-measure of VGG-16.

	Precision	Recall	*F*-measure
Healthy	0.81	0.75	0.78
Leukemia	**0.89**	**0.92**	**0.90**

**Table 4:** Precision, recall, and *F*-measure of the proposed convolutional neural network.

	Precision	Recall	*F*-measure
Healthy	0.81	0.70	0.75
Leukemia	**0.86**	**0.92**	**0.89**

**Table 5:** Precision, recall, and *F*1-score of machine learning classifiers for two classes of healthy and leukemia.

Method	Healthy			Leukemia		
	Precision	Recall	*F*1-score	Precision	Recall	*F*1-score
RF	**0.83**	0.57	**0.68**	0.81	**0.94**	**0.87**
LR	0.73	0.63	**0.68**	**0.83**	0.88	0.85
SVM	0.77	0.54	0.64	0.80	0.92	0.86
KNN	0.67	**0.66**	0.67	**0.83**	0.84	0.83
SGD	0.53	0.65	0.58	0.80	0.71	0.75
MLP	0.26	0.63	0.37	0.33	0.09	0.15

**Table 6:** Comparison of proposed networks with other machine learning-based classifiers.

Classifier	Training accuracy
VGG-16	0.9741
ResNet-50	0.9576
Proposed CNN	0.8579
RF	0.8172
LR	0.7988
SVM	0.7928
KNN	0.7789
SGD	0.6891
MLP	0.2733

**Table 7:** Results analysis and comparison of previous works.

Reference	Features employed	Classifier (s)	Test dataset	Technical environment	Optimal accuracy (%)
Thanh et al. [13]	Morphological features	CNN (with 7 layers)	357	MATLAB	96.60
Shafique and Tehsin [14]	Color features	AlexNet (with 8 layers)	306	MATLAB	99.50
Ahmed et al. [15]	Morphological features	CNN (with 6 layers)	511	Anaconda 3 with spider 3.3 and Python 3.6	88.25
Rehman et al. [8]	Morphological features	CNN (with 7 layers)	330	MATLAB	97.78
Kasani et al. [6]	Morphological features	An aggregated CNN (-)	2100	Keras package, with tensorflow	96.58
Kasani et al. [19]	Morphological features	CNN (-)	1454	Keras package, with tensorflow	96.17
Proposed approaches	CNN features	Three various CNNs	2506	Keras package, with tensorflow	**VGG-16**: 97.41, **ResNet-50**: 95.76, **proposed CNN**: 85.79

## Data Availability

The data generated or analyzed during this study are included in this published article.
